# Analysis of correlations between biomarkers assessed with swept-source OCT and OCT angiography in naïve patients with diabetic macular edema treated with aflibercept: a prospective study

**DOI:** 10.1186/s40942-025-00777-z

**Published:** 2025-12-17

**Authors:** Marcussi Palata Rezende, Fernanda Atoui Faria, Daniel Prado Beraldo, Julia Polido, Rubens Belfort Jr, Thiago Cabral

**Affiliations:** 1 Department of Ophthalmology, Federal University of São Paulo (UNIFESP), São Paulo, SP Brazil; 2 Clinica Oftalmo-Retina, Rua: Engenheiro Alfred Johann Liemert, 237, Sala 5A, Presidente Prudente, SP CEP:19.061-251 Brazil; 3 Department of Ophthalmology, Federal University of Espírito Santo (UFES), Vitoria, ES Brazil

**Keywords:** Correlations, Biomarkers, Diabetic macular edema, Aflibercept, Swept-source oct, Oct angiography, Choroidal thickness, Macular thickness

## Abstract

**Background:**

Swept-source OCT (SS-OCT) and OCT angiography (SS-OCTA) enable high-resolution assessment of retinal and choroidal biomarkers in diabetic macular edema (DME). However, prospective analyses of how these biomarkers correlate before and after anti-VEGF therapy in treatment-naïve eyes are limited. The aim of this study was to prospectively evaluate biomarker correlations following aflibercept treatment using state-of-the-art, high-resolution imaging with SS-OCT and SS-OCTA during 4 months of follow-up.

**Methods:**

This was a prospective interventional case series that included 28 eyes from 25 treatment-naïve patients with DME. All eyes received three monthly intravitreal aflibercept injections. Patients were reassessed one month after the loading phase (4-month visit). The evaluated biomarkers included best-corrected visual acuity (BCVA), central macular thickness (CMT), central choroidal thickness (CCT), vessel density (VD), and avascular area of the superficial plexus (AASP) and deep plexus (AADP). Pre- and posttreatment values were compared, and correlations were analyzed using Pearson’s or Spearman’s methods.

**Results:**

Significant changes in BCVA (0.7250 ± 0.23 to 0.3957 ± 0.21; *p* < 0.000), CMT μm (339.04 ± 66.19 to 265.21 ± 55.75; *p* < 0.000), CCT μm (221.71 ± 69.69 to 209.07 ± 70.92; *p* < 0.000), VD (17.90 ± 7.82 to 15.35 ± 5.80; *p* < 0.038), AASP μm^2^ (235,374 ± 91,299 to 157,326 ± 77,815; *p* < 0.000) and AADP μm^2^ (1996,335 ± 1,000,047 to 362,161 ± 277,225; *p* < 0.000) were detected. Very high correlations were observed for the CCT pre vs CCT post (*r* = 0.98; *p* < 0.001), and AADP pre vs AADP reduction (*r* = −0.93; *p* < 0.001), high correlation: VD pre vs VD reduction (*r* = −0.72; *p* < 0.001. In total, 2 correlations were very high, 1 high, 4 moderate, and 9 were low; all the correlations were statistically significant.

**Conclusions:**

Treatment-naïve DME eyes treated with aflibercept showed significant structural, vascular, and functional improvements, with several baseline biomarkers acting as potential predictive indicators of posttreatment outcomes. The very high correlation of CCT and AADP and the high correlation of VD suggest that SS-OCT and SS-OCTA can provide clinically useful information for identifying how patients will respond to treatment. These correlations support the role of SS-OCT and SS-OCTA not only as diagnostic tools but also as potential predictive indicators of therapeutic response, facilitating more personalized DME management in clinical practice. Notably, this is the first prospective study to evaluate correlations between SS-OCT and SS-OCTA biomarkers in naïve DME eyes treated with aflibercept.

## Introduction

Diabetic retinopathy (DR) is the leading cause of incident blindness in U.S. adults aged 20 to 74 years [1]. Diabetic macular edema (DME) is the major cause of vision loss in people with diabetes mellitus (DM) [2]. Together with proliferative DR, DME is a major source of vision loss in developed nations and accounts for much of the vision impairment in working-age adults [3]. The American Academy of Ophthalmology guideline (Diabetic Retinopathy PPP 2024) highlights that the treatment of DME is cost-effective, yielding substantial savings compared with the social costs of untreated visual impairment, while also reducing productivity losses and human suffering [4]. A systematic review by Cooper et al. (Diabetic Medicine, 2020) revealed that DME impairs daily activities, work, mobility, and psychological well-being, thereby amplifying the social and clinical impact of the disease [5].

Comparative evidence for antiangiogenic therapy in DME comes from the Diabetic Retinopathy Clinical Research Network (DRCR.net). In Protocol T, aflibercept, bevacizumab, and ranibizumab were compared head to head, and eyes starting with poorer vision ≤20/50 achieved greater gains in visual acuity (VA) with aflibercept. These data argue for individualized treatment because the response varies with each patient’s specific structural and functional characteristics. A five-year follow-up extension study confirmed that long-term outcomes are related to initial therapy choice and treatment maintenance over time [6].

Swept-source optical coherence tomography (SS-OCT) is an advanced retinal imaging technology that offers high resolution and speed; it operates at 100,000 A-scans per second, has a wavelength of 1050 nm, and achieves 8-μm axial resolution in tissue [7, 8]. These specifications allow automated quantification and three-dimensional (3D) reconstructions, and make the choroid–sclera boundary easier to trace, since the longer wavelength is less affected by dispersion at the retinal pigment epithelium (RPE). For this reason, SS-OCT is typically chosen when choroidal thickness is the variable of interest. Optical coherence tomography angiography (OCTA) adds a noninvasive, 3D view of the microvasculature by analyzing the decorrelation signal between B-scans in the same position and stratifying the retina into the superficial plexus, deep plexus, outer retina and choriocapillaris. SS-OCTA, which couples longer wavelengths with high-speed acquisition, further improves the rendering of deeper structures [7, 8].

Biomarkers are measurable variables that reflect pathophysiological mechanisms or biological responses to therapeutic interventions, and have been widely used in clinical studies. Their use can reduce sample size, duration, and costs of clinical trials, especially when primary outcomes are rare or require prolonged follow-up. The main function of a biomarkers in clinical research is to act as objective and quantifiable indicators of a normal biological process, a pathological condition, or the response to a therapeutic intervention. Biomarkers can be used for several purposes, including early diagnosis, risk stratification, monitoring disease progression, and evaluating evaluating treatment efficacy. In clinical trials, they are often used as intermediate endpoints, with the aim of predicting final clinical outcomes, enabling faster and less costly therapeutic decision-making. When well validated, biomarkers support causal inference, aid in prognostic modeling, and improve the accuracy of clinical estimates, directly contributing to the practice of medicine [9].

The use of OCT and OCT angiography to identify structural and vascular biomarkers has become central to evaluating DME, since these methods allow precise follow up of the disease course and response to therapy [10]. A range of metrics, such as central macular thickness (CMT), choroidal vascularity index (CVI), central choroidal thickness (CCT), foveal avascular zone (FAZ), vessel density (VD), deep capillary plexus (DCP), and the superficial capillary plexus (SCP), have been explored as candidate biomarkers. Together, these indicators capture the microvascular and tissue level alterations that accompany DME and support a more tailored approach to diagnosis, risk assessment, and treatment planning. When interpreted in combination, biomarkers also increase our undestanding of DME pathophysiology and inform the design of better therapeutic strategies. For these reasons, quantitative evaluation with OCT and OCTA has become an indispensable component of clinical practice and research in DR [10].

A correlation is a statistical measure that assesses the existence, direction, and strength of a linear association between two continuous variables. As described by Mukaka (2012) [11], a correlation coefficient is a dimensionless quantity that ranges from −1 (perfect negative correlation) to + 1 (perfect positive correlation), with 0 indicating no linear relationship. The two main types are: the Pearson correlation coefficient, which is used when both variables have a normal distribution, and the Spearman coefficient, which is used when one or both variables do not follow a normal distribution or when the data are of the ordinal type (i.e., classifications or rankings). The interpretation of the magnitude of the correlation follows a convention: values between 0.00 and 0.19 indicate negligible correlation; 0.20–0.39, weak; 0.40–0.59, moderate; 0.60–0.79, strong; and 0.80–1.00, very strong (Table 1). In scientific studies, especially in clinical research with biomarkers, identifying relevant correlations can reveal pathophysiological interactions between variables, help understand the mechanisms of therapeutic action, and suggest prognostic or predictive targets. In clinical practice, these correlations guide personalized decisions, optimize treatments, and anticipate clinical responses, improving the management of complex diseases [11]. Hence, finding correlations between biomarkers in diseases that we will treat is important, which is the main objective of our study.

**Table 1:** Rule of thumb for interpreting the size of a correlation coefficient

Size of Correlation	Interpretation
0.90 to 1.00 (−0.90 to −1.00)	Very high positive (negative) correlation
0.70 to 0.90 (−0.70 to −0.90)	High positive (negative) correlation
0.50 to 0.70 (−0.50 to −0.70)	Moderate positive (negative) correlation
0.30 to 0.50 (−0.30 to −0.50)	Low positive (negative) correlation
0.00 to 0.30 (0.00 to −0.30)	Negligible correlation

Adapted from Mukaka MM. Statistics Corner: A guide to appropriate use of correlation coefficient in medical research. Malawi Med J. 2012 Sep;24(3):69–71

Despite the widespread clinical use of imaging biomarkers and advances in OCT and OCTA based biomarkers, studies that systematically explore their correlations throughout treatment are still scarce, especially in treatment-naïve patients, and prospective evidence on how retinal and choroidal metrics interact during anti-VEGF therapy in DME patients is lacking.

This study is the first to prospectively evaluate the correlations between retinal and choroidal biomarkers obtained by SS-OCT and SS-OCTA, before and after treatment with a loading dose of aflibercept 2 mg (three monthly doses) in naïve-patients with DME. These findings improve our understanding of the structural and vascular mechanisms involved in therapeutic response. In this context, our study expands this perspective by utilizing state-of-the-art imaging technology to investigate the correlations between these biomarkers. We also aim to contribute to a more personalized approach to DME treatment by providing new insights into the impact of antiangiogenic therapy on the choroidal and retinal microenvironments, thus filling a critical gap and highlighting the potential of SS-OCT and SS-OCTA biomarkers to guide personalized treatment.

## Objective

The aim of this study was to evaluate treatment-naïve patients with DME via SS-OCT and SS-OCTA, before treatment, following a loading dose regimen of aflibercept, and after a 4-month follow-up period. The primary objectives were as follows:

- To determine whether statistically significant correlations exist between pre and posttreatment biomarker values: best-corrected visual acuity (BCVA), CMT, CCT, AASP, AADP, and VD.
- To analyze the variations in structural and vascular biomarkers of the choroid and macula before and after treatment with a loading dose.

## Methods

Participants were recruited at Clínica Oftalmo-Retina, Presidente Prudente, São Paulo, Brazil. The investigation followed a prospective, noncomparative interventional case-series design. Eligibility required a diagnosis of diffuse DME and the absence of any previous treatment for the condition.

### A-) inclusion criteria

Patients were eligible for the study if they met the following conditions:

1. Adults ≥18 years with type 2 diabetes and diffuse DME who were treatment-naïve for DME were included.
2. Loading injections were delivered by a single ophthalmologist throughout the study period.
3. Comprehensive records, including demographics, duration of diabetes, current medications, intraocular pressure (IOP), BCVA, and SS-OCT/SS-OCTA imaging before and after therapy, were available.
4. BCVA within 20/25 to 20/200, obtained using the Early Treatment Diabetic Retinopathy Study (ETDRS) protocol and expressed as Snellen values.
5. Signed informed consent documents that were stored with the patient chart

### B-) exclusion criteria

Patients were excluded if any of the following applied:

1. Receipt of any prior intravitreal anti-VEGF therapy.
2. Incomplete loading phase, defined as a failure to receive all three scheduled intravitreal injections for any cause.
3. Coexisting retinal or optic nerve disease that could confound the outcomes, including age-related macular degeneration, glaucoma, vitreoretinal interface disorders (epiretinal membrane, macular hole, proliferative vitreomaculopathy) or inherited retinal dystrophies.
4. A history of ocular surgery, such as retinal detachment repair or glaucoma procedures, likely to affect macular status or IOP control.

A control group was not included given the study’s purpose and ethical constraints. The project followed a prospective interventional design that measured retinal biomarkers in patients with diffuse DME at baseline and after a standardized loading regimen. Creating an arm without treatment simply for comparison would deny participants access to therapy regarded as standard care in this setting and would contravene principles outlined in the Declaration of Helsinki. By using each eye as its own comparator across time, the study preserved internal validity for the targeted outcomes and, at the same time, met the ethical requirements to treat eligible patients.

#### Ethical Committee approval

This research followed Resolution 196/96 of the National Health Council (Ministry of Health, Brazil) and was conducted in accordance with the principles of the Declaration of Helsinki. The study protocol underwent ethical review and received formal approval from the Ethics Committee of the Hospital Regional do Câncer da Santa Casa de Misericórdia de Presidente Prudente, São Paulo, under CAAE: 19386619.10000.8247.

## Data collection and examination techniques

From November 2019 to January 2022, individuals who fulfilled the eligibility requirements and provided written consent were prospectively included.

Once diffuse DME was confirmed and the core dataset was obtained, assessments covered demographic age, sex, duration of diabetes, systemic and ocular antecedents, diabetes classification type 1 or 2, Diabetic Retinopathy Classification, and a comprehensive ophthalmologic examination. At baseline, the protocol included medical history, BCVA, IOP, slit-lamp biomicroscopy, dilated fundus examination, seven-field color photography, red-free imaging, fundus autofluorescence, fluorescein angiography, swept-source OCT, and swept-source OCT angiography. The entire panel of tests was repeated four weeks after the loading phase was completed. BCVA was obtained using Snellen charts and converted to the logarithm of the minimum angle of resolution (logMAR) for statistical analysis.

Retinal and choroidal imaging was performed with SS-OCT and SS-OCTA on a DRI-OCT Triton system Topcon, Tokyo, Japan. Automated layer segmentation was applied and the results were subsequently reviewed. SS-OCT used 7 × 7 mm cubes, and SS-OCTA used 4.5 × 4.5 mm scans. Retinal thickness was defined as the distance from the vitreoretinal interface to the RPE. Choroidal thickness was defined as the distance from the border of the RPE to the chorio-scleral boundary. The device’s calibration tools provided these distance measurements. CMT and CCT were defined as the mean thickness within the central 1000 µm of the ETDRS grid using the macular and choroidal maps provided by the SS-OCT software. Each segmentation line, namely, the vitreoretinal interface, RPE, and chorio-scleral boundary, was inspected and manually corrected when needed. SS-OCTA outputs included the following: A-) VD: automatically computed by the software Imaginet 6, representing total retinal VD combining the SCP and DCP. B-) FAZ in the superficial and deep layers, recorded as AASP and AADP, respectively. These areas were traced manually by an experienced ophthalmologist and verified by a second reader because the software does not generate them automatically. Image acquisition and grading were masked. Two independent ophthalmologists analyzed all the scans (SS-OCT and SS-OCTA), and disagreements were adjudicated by a third reviewer.

A trained imaging technician, unaware of both the study procedures and each patient’s treatment status, performed all the SS-OCT and SS-OCTA scans. Examinations were systematically conducted appoximately 10:00 a.m. to mitigate diurnal changes in choroidal thickness that can affect quantitative measurements [12, 13].

Evaluation of DME on SS-OCT included the detection of subretinal fluid and/or intraretinal cysts, and/or RPE detachment and the confirmation of CMT greater than 250 µm [14].

This study used a predefined care path with two steps. The initial step involved a loading regimen of aflibercept administered intravitreally once per month for three consecutive months. The second step was a post-loading evaluation performed one month after the third injection; thus, the outcomes were analyzed approximately four months after baseline. Imaging with SS-OCT and SS-OCTA was performed at baseline and repeated one month after the third injection to quantify anatomical and microvascular changes. All procedures were carried out by a single senior ophthalmologist in a surgical suite. After completion of the loading course, participants continued under surveillance and received further treatment if signs of DME remained. Injections were standardized as follows: aflibercept 0.05 ml 2 mg; concentration 40 mg/ml; injected via pars plana in the superotemporal quadrant; 30-gauge needle 0.3 × 13 mm; and entry site 3.5 mm posterior to the limbus.

### Statistical analysis

#### Correlations

A Gaussian distribution test was performed to verify a normal distribution in the groups analyzed. Correlation analyses between continuous variables were performed using Pearson’s correlation when the groups presented a normal distribution and using Spearman’s correlation for groups without a normal distribution. And ninety-five percent confidence intervals for correlation coefficients were calculated using Fisher’s z-transformation.

#### Pre- and posttreatment comparisons

A Gaussian distribution test was performed to verify a normal distribution in the groups analyzed. Mean and standard deviation analyses were performed, and Student’s t test was performed to compare the pre- and posttreatment data that presented a normal distribution. When a normal distribution was not found, the Wilcoxon test was used to compare pre- and posttreatment data.

#### Patient characteristics

The population characteristics, such as age and sex, are presented in tables with mean and standard deviation measurements.

To perform the statistical analysis, IBM SPSS v0.24 software was used and a significance level of 5% was adopted for all analyses.

## Results

### A-) General results

A total of 28 eyes from 25 individuals with treatment-naïve DME were analyzed following a standardized loading regimen. The average age was 66.79 ± 10.26 years. The sample included 12 men (48%) and 13 women (52%). Laterality was balanced, with 13 right eyes (46.42%) and 15 left eyes (53.58%). With respect to lens status, 10 eyes were phakic (35.72%), and 18 were pseudophakic (64.28%), as summarized in Table 2.

**Table 2:** Description of clinical and personal characteristics of participants

Number of patients (N) Number of eyes (N)	25 28
Age (years)	66.79 ± 10.26
Sex	
Male	48.00% (12)
Female	52.00% (13)
Eye	
Right	46.42% (13)
Left	53.58% (15)
Lens status	
Phakic	35.72% (10)
Pseudophakic	64.28% (18)

Outcomes were compared at baseline and 1 month after the loading phase for BCVA, CMT, CCT, IOP, VD, AASP and AADP. All the variables demonstrated significant posttreatment changes except for IOP, which remained unchanged (Table 3). BCVA improved from 0.7250 ± 0.23 to 0.3957 ± 0.21 logMAR (*p* < 0.001). Structurally, the CMT decreased from 339.04 ± 66.19 µm to 265.21 ± 55.75 µm (*p* < 0.001), and the CCT decreased from 221.71 ± 69.69 µm to 209.07 ± 70.92 µm (*p* < 0.001). The microvascular indices also shifted, with VD decreasing from 17.90 ± 7.82 to 15.35 ± 5.80 (*p* = 0.038), AASP decreasing from 235,374 ± 91,299 µm^2^ to 157,326 ± 77,815 µm^2^ (*p* < 0.001), and AADP decreasing from 996,335 ± 1,000,047 µm^2^ to 362,161 ± 277,225 µm^2^ (*p* < 0.001). According to the Diabetic Retinopathy Classification by the ETDRS, 4 of the 28 eyes (14.29%) presented moderate nonproliferative diabetic retinopathy, whereas 24 eyes (85.71%) presented severe nonproliferative disease.

**Table 3:** Description of pre and posttreatment groups

Variable	Pre	Post	P-value
BCVA (logMAR)	0.7250 ± 0.23	0.3957 ± 0.21	0.000*
CMT (µm)	339.04 ± 66.19	265.21 ± 55.75	0.000*
CCT (µm)	221.71 ± 69.69	209.07 ± 70.92	0.000*
IOP (mmHg)	12.68 ± 1.30	12.79 ± 1.44	0.641
VD	17.90 ± 7.82	15.35 ± 5.80	0.038*
AASP (µm^2^)	235,374 ± 91,299	157,326 ± 77,815	0.000*
AADP (µm^2^)	996,335 ± 1,000,047	362,161 ± 277,225	0.000*

P-value for the Wilcoxon signed-rank test. Values less than 0.05 indicate a statistically significant difference between groups * **p < 0.05** BCVA: best-corrected visual acuity; logMAR: logarithm of the minimum angle of resolution; CMT: central macular thickness; CCT: central choroidal thickness; IOP: intraocular pressure; VD: vessel density; AASP: avascular area of the superficial plexus; AADP: avascular area of the deep plexus

**Fig. 1:**
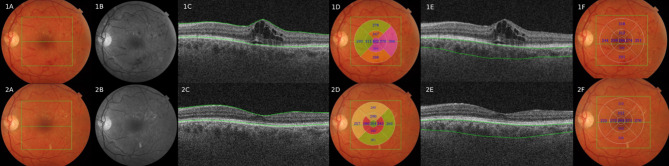
Multimodal assessment with SS-OCT of a patient participating in this study, who received treatment with a loading dose of aflibercept (40 mg/ml) 0,05 ml/2 mg. 1A: pretreatment color retinography: presence of diabetic retinopathy (microaneurysms and cotton wool exudates) and the central B-scan. 2A: posttreatment color retinography. All images that begin with number 1 correspond to pretreatment, and all images that begin with number 2 correspond to posttreatment. Image 1B: red free pretreatment: presence of microaneurysms and cotton-wool exudates. 2B: red free posttreatment. 1C: central B-Scan SS-OCT: delimitation of macular thickness, where we observe the presence of diabetic macular edema. 2C: posttreatment: central B-scan without macular edema. 1D: ETDRS map with central macular thickness of 402 µm, and 2C: posttreatment with central macular thickness of 254 µm. 1E: B-Scan with choroidal thickness delimitation, and 2E: choroidal thickness posttreatment. 1F: central choroidal thickness of 286 µm pretreatment. And 2E: 293 µm

**Fig. 2:**
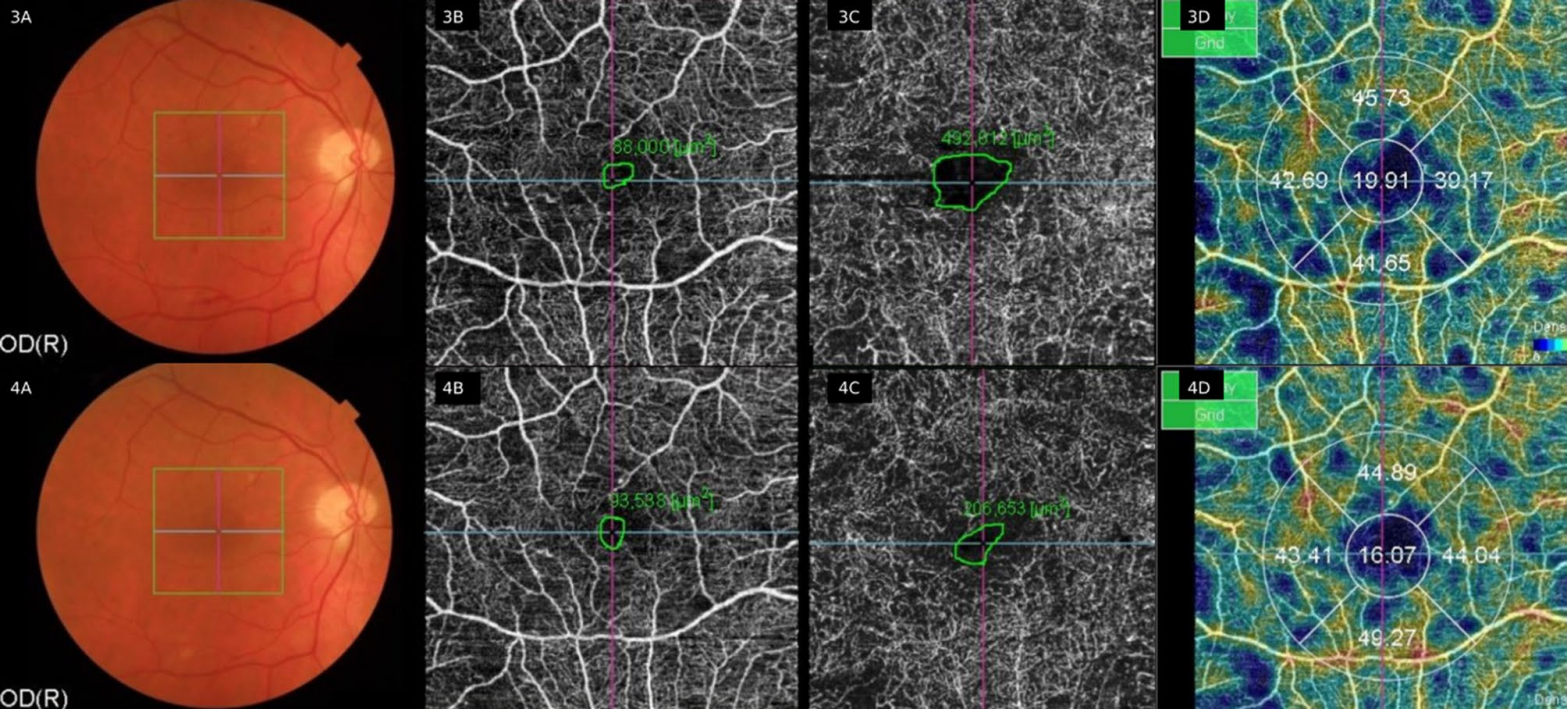
1A: multimodal assessment with SS-OCT-Angiography of another patient participating in this study. 3A: pretreatment: color retinography showing the 4.5x4.5 mm square of the octa scans. 4A: posttreatment color retinography. 3B: avascular area of the superficial plexus pretreatment: 88,000 µm^2^, and 4B: posttreatment with 93,538 µm^2^. Image 3C: avascular area of the deep plexus pretreatment: 492,012 µm^2^, and 4C: posttreatment with reduction to 206,653 µm^2^. 3D: vessel density map = 19.91 corresponding to the central. 4D: shows decreased central vessel density = 16.07

Figures 1 and 2 show images of patients before and after treatment with a loading dose of aflibercept. The SS-OCT analysis of structural biomarkers (CMT and CCT) for a patient included in this study is shown in Fig. 1, and the analysis of vascular biomarkers with SS-OCTA (AASP, AADP and VD) is shown in Fig. 2 for another patient in this study. Both figures show the biomarker measurements before and after treatment with a loading dose of aflibercept.

### B-) Correlation analysis

In our study, we were able to demonstrate several statistically significant correlations between the various pre- and posttreatment biomarkers provided by SS-OCT and SS-OCTA. Among the main correlations with statistical significance, we can highlight the following (Table 4):

**Table 4:** Correlation coefficients (r), 95% confidence intervals, and p-values between structural, vascular, and functional biomarkers derived from SS-OCT and SS-OCTA in treatment-naïve eyes with diabetic macular edema treated with aflibercept.

Biomarker 1	Biomarker 2	r	95% CI (lower)	95% CI (upper)	p-value	Interpretation*
1-) *CMT pre*	*CMT post*	0.46	0.10	0.71	0.012	Low positive
2-) *CMT pre*	*CMT reduction*	-0.61	-0.80	-0.31	<0.001	Moderate negative
3-) *CMT pre*	*BCVA post*	0.38	0.01	0.66	0.046	Low positive
4-) *CMT pre*	*AADP pre*	0.51	0.17	0.74	0.005	Moderate positive
5-) *CMT pre*	*AADP post*	0.41	0.04	0.68	0.028	Low positive
6-) *CMT pre*	*AADP reduction*	-0.47	-0.72	-0.12	0.011	Low negative
7-) *CCT pre*	*CCT post*	0.98	0.96	0.99	<0.001	Very high positive
8-) *AADP pre*	*AADP post*	0.48	0.13	0.72	0.009	Low positive
9-) *AADP pre*	*AADP reduction*	-0.93	-0.97	-0.85	<0.001	Very high negative
10-) *AADP pre*	*AASP pre*	0.44	0.08	0.70	0.018	Low positive
11-) *AADP pre*	*AASP post*	0.37	-0.00	0.65	0.048	Low positive
12-) *AASP pre*	*AADP post*	0.39	0.02	0.67	0.037	Low positive
13-) *AASP pre*	*AASP reduction*	-0.65	-0.82	-0.37	<0.001	Moderate negative
14-) *VD pre*	*VD post*	0.62	0.32	0.81	<0.001	Moderate positive
15-) *VD pre*	*VD reduction*	-0.72	-0.86	-0.47	<0.001	High negative
16-) *VD pre*	*BCVA reduction*	0.38	0.01	0.66	0.042	Low positive

*Interpretation of correlation size according to Mukaka (2012): very high (|r| ≥0.90), high (0.70–0.89), moderate (0.50–0.69), low (0.30–0.49). Values are Pearson’s or Spearman’s coefficients, as appropriate. “Change” refers to post- minus pre-treatment values (negative values indicate reductions). 95% confidence intervals were computed using Fisher’s z transformation with n=28. Wider intervals reflect the limited sample size and should be interpreted with caution. Pearson’s or Spearman’s coefficients were used as appropriate

BCVA: best-corrected visual acuity; logMAR: logarithm of the minimum angle of resolution; CMT: central macular thickness; CCT: central choroidal thickness; IOP: intraocular pressure; VD: vessel density; AASP: avascular area of the superficial plexus; AADP: avascular area of the deep plexus. CI: confidence interval. r = correlation coefficient

**Table 5:** All Correlations between the biomarkers (SS-OCT and SS-OCTA).

	CMT pre (µm)	CMT Post (µm)	CMT Reduction (µm)	CMT Reduction (%)	CCT Pre (µm)	CCT Post (µm)	CCT Reduction (µm)	CCT Reduction (%)	IOP Pre (mmHg)	IOP Post (mmHg)	IOP Reduction (mmHg)	IOP Reduction (%)	AADP Pre (µm^2^)	AADP Post (µm^2^)	AADP Reduction (µm^2^)	AADP Reduction (%)	AASP Pre (µm^2^)	AASP Post (µm^2^)	AASP Reduction (µm^2^)	AASP Reduction (%)	VD Pre	VD Post	VD Reduction	VD Reduction (%)	BCVA Pre (logMAR)	BCVA Post (logMAR)	BCVA Reduction (logMAR)	BCVA Reduction (%)
**CMT pre (µm)**																												
**CMT Post (µm)**	0.46																											
	**0.012**																											
**CMT Reduction (µm)**	-0.61	0.25																										
	**0.000485**																											
**CMT Reduction (%)**	-0.41	0.46																										
	0.028	**0.0140**																										
**CCT Pre (µm)**	-0.17	-0.35	-0.02	-0.09																								
	0,373	0,0650	0,8850	0,6240																								
**CCT Post (µm)**	-0.17	-0.34	-0.01	-0.11	0.98																							
	0.373	0.071	0.944	0.571	**0.000000000**																							
**CCT Reduction (µm)**	-0.11	-0.17	0.00	-0.08	-0.01	0.18																						
	0.547	0.377	0.977	0.688	0.960	0.344																						
**CCT Reduction (%)**	-0.16	-0.24	0.00	-0.07	0.16	0.34																						
	0.404	0.205	0.964	0.722	0.417	0.072																						
**IOP Pre (mmHG)**	-0.17	-0.34	-0.08	-0.18	-0.19	-0.19	-0.01	-0.04																				
	0.364	0.070	0.682	0.352	0.329	0.331	0.936	0.817																				
**IOP Post (mmHg)**	0.18	0.03	-0.04	-0.14	-0.11	-0.09	0.06	0.36	0.30																			
	0.343	0.852	0.842	0.455	0.575	0.627	0.758	0.857	0.119																			
**IOP Reduction (mmHg)**	0.33	0.36	0.03	0.11	-0.06	-0.02	0.18	0.15	0.55	0.59																		
	0.086	0.057	0.867	0.546	0.751	0.901	0.334	0.421	**0.002**	**0.001**																		
**IOP Reduction (%)**	0.34	0.32	0.00	0.01	0.02	0.00	0.15	0.13	0.47	0.66																		
	0.072	0.094	0.986	0.959	0.892	0.971	0.441	0.479	**0.010**	**0.000126**																		
**AADP Pre (µm**^**2**^**)**	0.51	0.16	-0.40	-0.33	-0.11	-0.12	-0.33	-0.37	0.09	0.14	0.00	0.04																
	**0.005**	0.401	**0.034**	0.086	0.549	0.526	0.078	0.052	0.637	0.458	0.964	0.809																
**AADP Post (µm**^**2**^**)**	0.41	0.21	-0.15	-0.04	0.04	-0.02	-0.55	-0.49	0.14	0.08	-0.08	-0.04	0.48															
	**0.028**	0.283	0.426	0.834	0.836	0.910	**0.002**	**0.007**	0.451	0.676	0.677	0.831	**0.009**															
**AADP Reduction (µm**^**2**^**)**	-0.47	-0.15	0.37	0.31	0.16	0.15	0.18	0.24	0.01	-0.06	-0.03	-0.06	-0.93	-0.19														
	**0.011**	0.443	**0.048**	0.103	0.413	0.441	0.344	0.203	0.942	0.728	0.863	0.743	0.000000003	0.330														
**AADP Reduction (%)**	-0.27	-0.04	0.32	0.31	0.16	0.12	-0.12	-0.03	0.05	0.00	-0.01	-0.02	-0.62	0.33														
	0.150	0.835	0.097	0.098	0.393	0.527	0.540	0.870	0.786	0.972	0.829	0.914	**0.000343**	0.085														
**AASP Pre (µm**^**2**^**)**	0.10	-0.14	-0.12	-0.19	-0.03	-0.06	-0.14	-0.14	0.04	-0.22	-0.24	-0.21	0.44	0.39	-0.38	-0.19												
	0.612	0.452	0.537	0.322	0.873	0.760	0.448	0.477	0.842	0.261	0.219	0.279	**0.018**	**0.037**	**0.043**	0.312												
**AASP Post (µm**^**2**^**)**	0.29	-0.06	-0.45	-0.44	-0.12	-0.13	-0.05	-0.08	0.04	0.25	0.06	0.19	0.37	0.18	-0.35	-0.25	0.37											
	0.125	0.763	**0.016**	**0.019**	0.532	0.501	0.766	0.673	0.810	0.186	0.756	0.334	**0.048**	0.352	0.067	0.188	0.052											
**AASP Reduction (µm**^**2**^**)**	0.15	0.12	-0.25	-0.17	-0.07	-0.05	0.09	0.06	-0.01	0.37	0.28	0.32	0.07	-0.18	0.05	-0.01	-0.65	0.46										
	0.430	0.518	0.200	0.376	0.723	0.799	0.632	0.739	0.937	0.052	0.150	0.096	0.723	0.352	0.797	0.927	**0.000162**	**0.014**										
**AASP Reduction (%)**	0.15	-0.05	-0.40	-0.46	0.04	0.03	0.02	0.03	0.00	0.37	0.30	0.31	0.15	-0.07	-0.14	-0.15	-0.21	0.78										
	0.428	0.793	**0.033**	**0.013**	0.814	0.873	0.918	0.849	0.981	**0.048**	0.119	0.103	0.425	0.723	0.463	0.425	0.273	**0.0088264**										
**VD Pre**	0.09	0.19	0.11	0.18	-0.10	-0.09	0.08	0.06	-0.43	0.03	0.48	0.37	-0.19	-0.22	0.08	-0.70	-0.19	-0.19	0.02	-0.04								
	0.621	0.324	0.547	0.359	0.582	0.646	0.683	0.761	**0.020**	0.844	**0.010**	**0.048**	0.319	0.258	0.678	0.725	0.327	0.309	0.914	0.827								
**VD Post**	0.27	0.55	0.19	0.24	-0.16	-0.14	0.11	0.03	-0.54	0,10	0.61	0.52	-0.01	0.13	0.04	0.09	-0.23	-0.10	-0.06	-0.09	0.62							
	0.150	**0.002**	0.329	0.070	0.396	0.473	0.533	0.866	**0.002**	0.596	**0.001**	**0.004**	0.954	0.480	0.831	0.650	0.908	0.587	0.740	0.630	**0.000417**							
**VD Reduction**	0.01	0.18	0.05	0.08	0.09	0.15	-0.03	0.00	0.19	0.04	-0.05	-0.10	0.19	0.25	-0.05	0.09	0.25	0.15	-0.05	0.05	-0.72	-0.01						
	0.943	0.357	0.797	0.674	0.644	0.439	0.856	0.980	0.324	0.806	0.770	0.604	0.332	0.185	0.781	0.631	0.184	0.425	0.786	0.771	**0.000012**	0.924						
**VD Reduction (%)**	0.05	0.27	0.10	0.14	0.10	0.17	-0.03	0.00	0.10	0.03	0.00	-0.07	0.17	0.28	-0.02	0.11	0.23	0.07	-0.10	0.00	-0.56	0.17						
	0.771	0.156	0.597	0.455	0.583	0.380	0.859	0.974	0.604	0.880	0.974	0.715	0.379	0.137	0.889	0.559	0.233	0.693	0.606	0.963	**0.002**	0.370						
**BCVA Pre (logMAR)**	0.20	-0.19	-0.47	-0.36	0.33	0.30	-0.03	0.04	-0.02	-0.40	-0.33	-0.33	-0.06	0.00	0.04	-0.01	0.20	0.15	-0.05	0.08	-0.22	-0.27	0.09	0.04				
	0.304	0.311	**0.010**	0.056	0.078	0.116	0.864	0.836	0.882	**0.031**	0.080	0.083	0.744	0.971	0.812	0.927	0.287	0.438	0.788	0.656	0.246	0.155	0.634	0.820				
**BCVA Post (logMAR)**	0.38	0.20	-0.22	-0.09	0.13	0.10	-0.23	-0.17	-0.29	-0.16	0.08	0.08	0.07	0.45	0.04	0.23	0.20	0.14	-0.10	0.04	0.15	0.19	-0.03	0.04	0.36			
	**0.046**	0.306	0.252	0.627	0.488	0.604	0.223	0.372	0.125	0.408	0.667	0.685	0.710	**0.016**	0.819	0.229	0.292	0.465	0.581	0.827	0.437	0.332	0.881	0.805	0.055			
**BCVA Reduction (logMAR)**	0.12	0.45	0.29	0.35	-0.09	-0.10	-0.22	-0.24	-0.30	0.09	0.29	0.26	0.16	0.34	-0.08	0.11	-0.03	-0.08	-0.03	-0.08	0.38	0.41	-0.16	-0.02	-0.48	0.50		
	0.535	**0.015**	0.122	0.066	0.634	0.613	0.242	0.211	0.109	0.633	0.128	0.177	0.398	0.076	0.656	0.548	0.849	0.654	0.852	0.686	**0.042**	**0.027**	0.391	0.892	**0.010**	**0.007**		
**BCVA Reduction (%)**	0.24	0.39	0,12	0.22	-0.07	-0.13	-0.28	-0.21	-0.27	0.02	0.26	0.19	0.12	0.43	0.00	0.20	0.08	-0.03	-0.11	-0.06	0.42	0.30	-0.14	0.00	-0.18	0.79	0.91	
	0.219	**0.038**	0.528	0.260	0.689	0.499	0.143	0.276	0.157	0.916	0.172	0.330	0.535	**0.021**	0.967	0.308	0.651	0.867	0.568	0.761	**0.024**	0.113	0.450	0.966	0.334	**0.00533**	**0.00000002**	

BCVA: best-corrected visual acuity; logMAR: logarithm of the minimum angle of resolution; CMT: central macular thickness; CCT: central choroidal thickness; IOP: intraocular pressure; VD: vessel density; AASP: avascular area of the superficial plexus; AADP: avascular area of the deep plexus

**Graph. 1:**
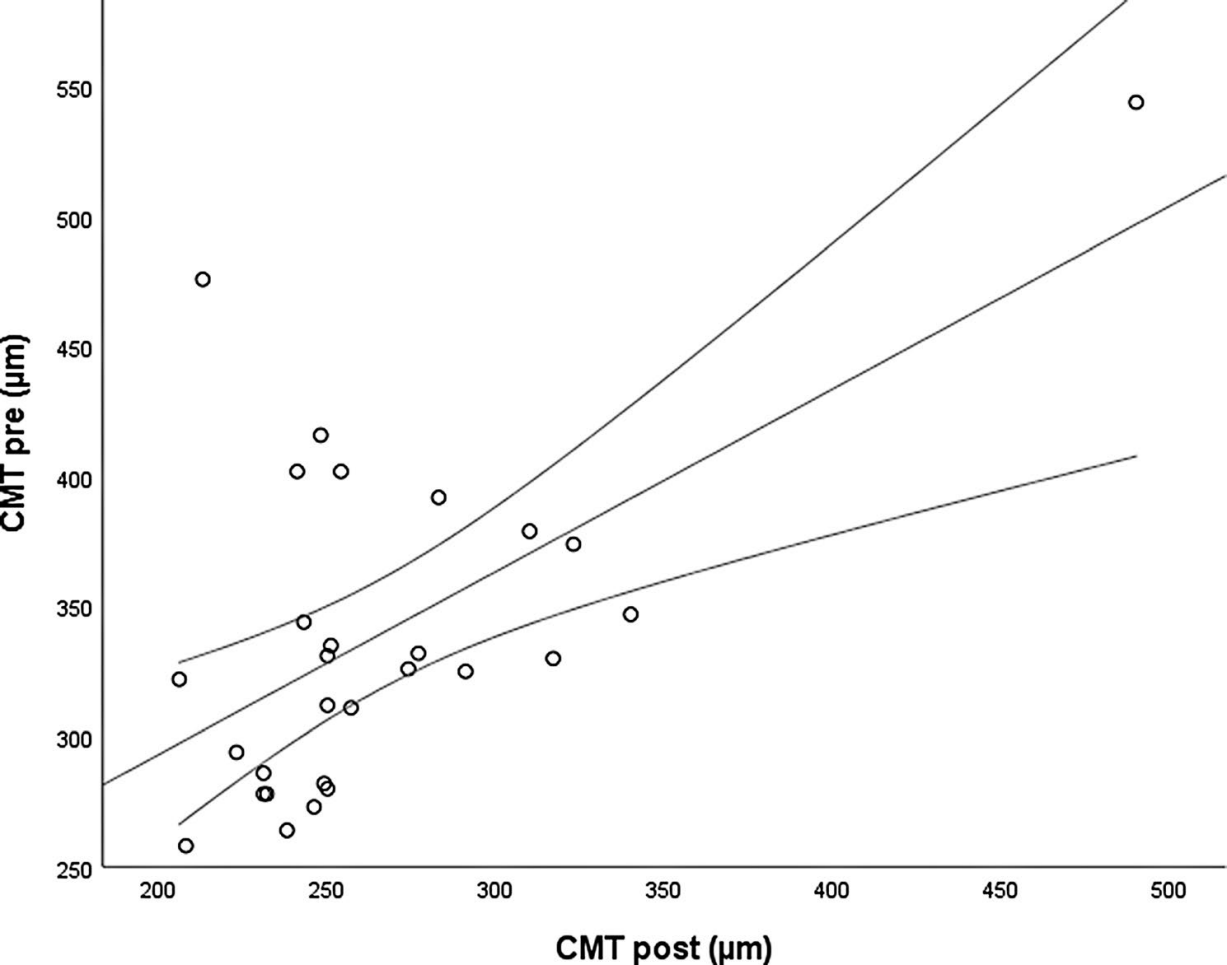
CMT pre (µm): central macular thickness pretreatment, CMT post (µm): central macular thickness posttreatment

**Graph. 2:**
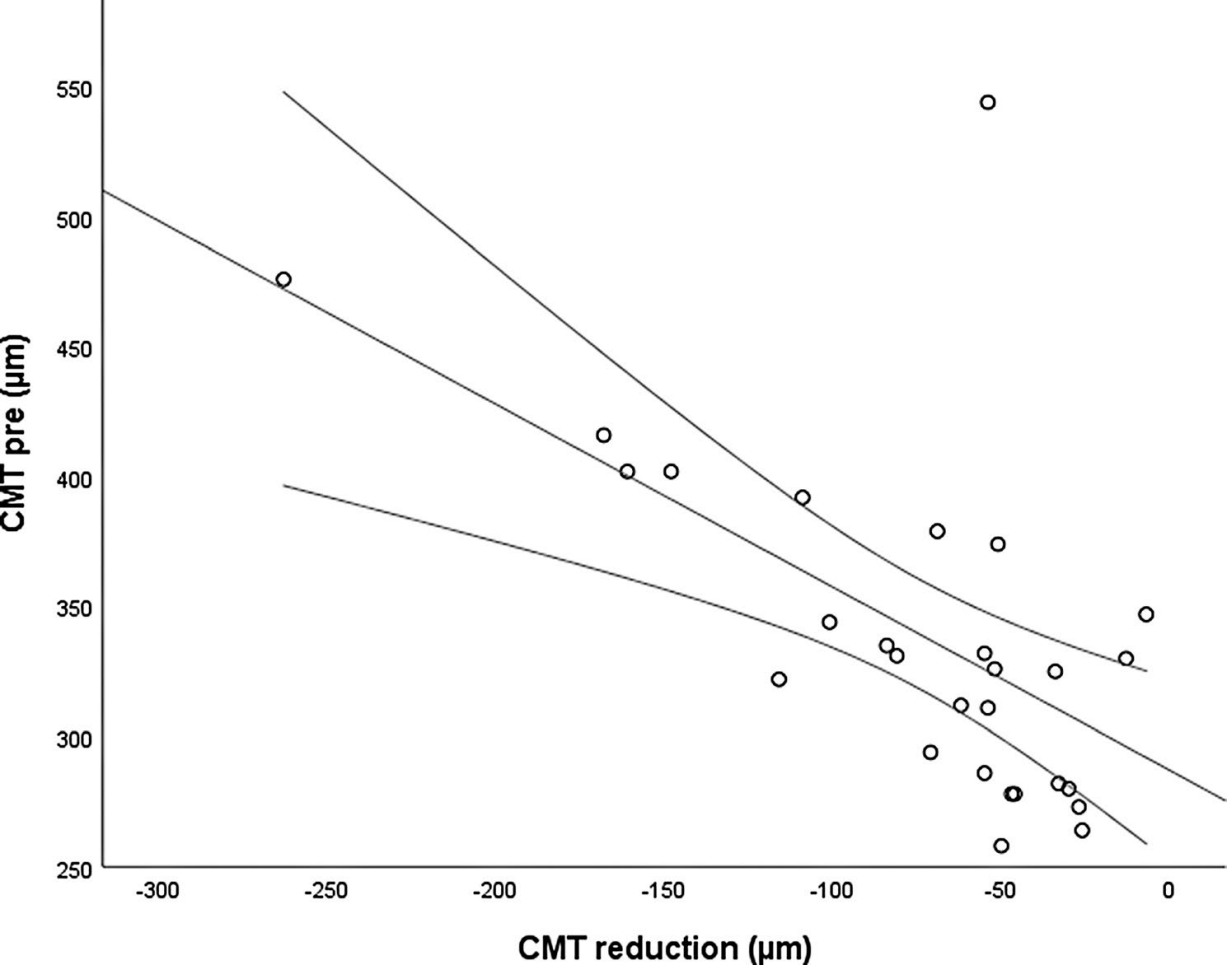
CMT pre (µm): central macular thickness pretreatment, CMT reduction (µm): reduction of central macular thickness

**Graph. 3:**
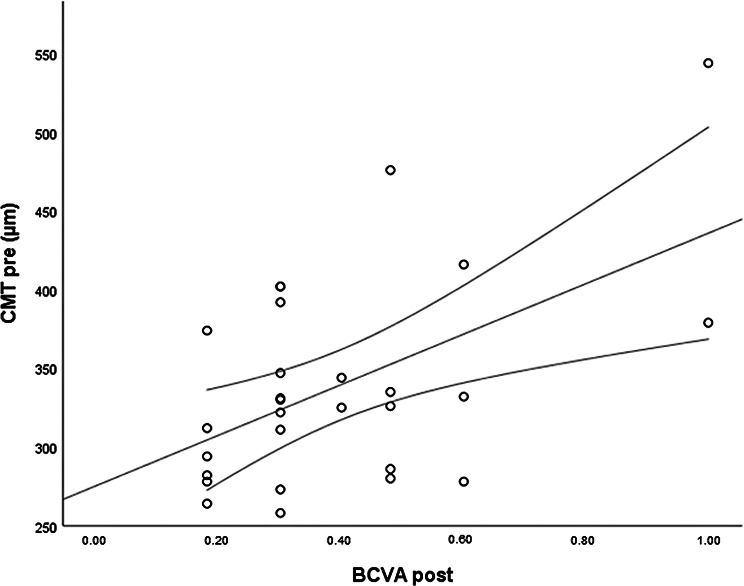
CMT pre (µm): central macular thickness pretreatment, BCVA: best-corrected visual acuity posttreatment

**Graph. 4:**
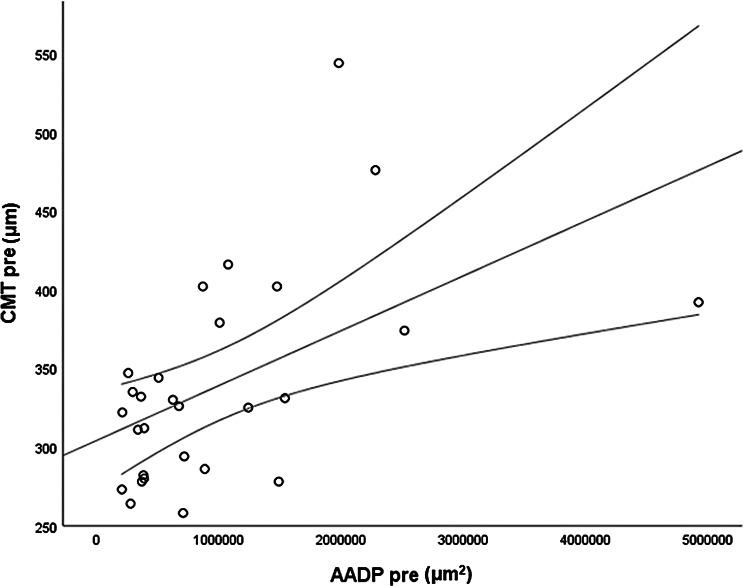
CMT pre (µm): central macular thickness pretreatment, AADP pre (µm2): avascular area of the deep plexus pretreatment

**Graph. 5:**
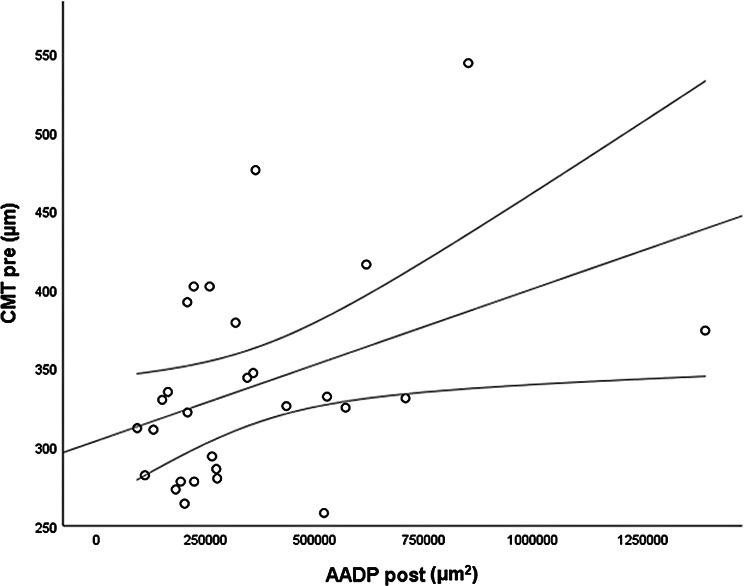
CMT pre (µm): central macular thickness pretreatment, AADP post (µm^2^): avascular area of the deep plexus posttreatment

**Graph. 6:**
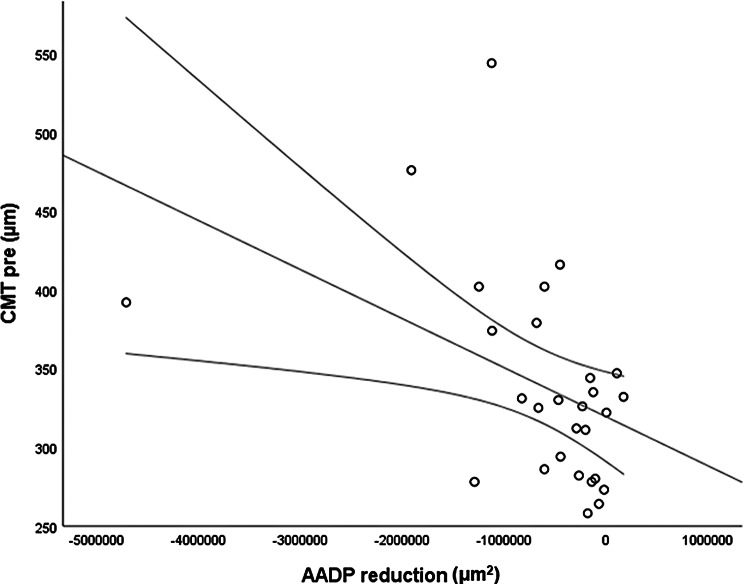
CMT pre (µm): central macular thickness pretreatment, AADP reduction (µm2): reduction of the avascular area of the deep plexus

**Graph. 7:**
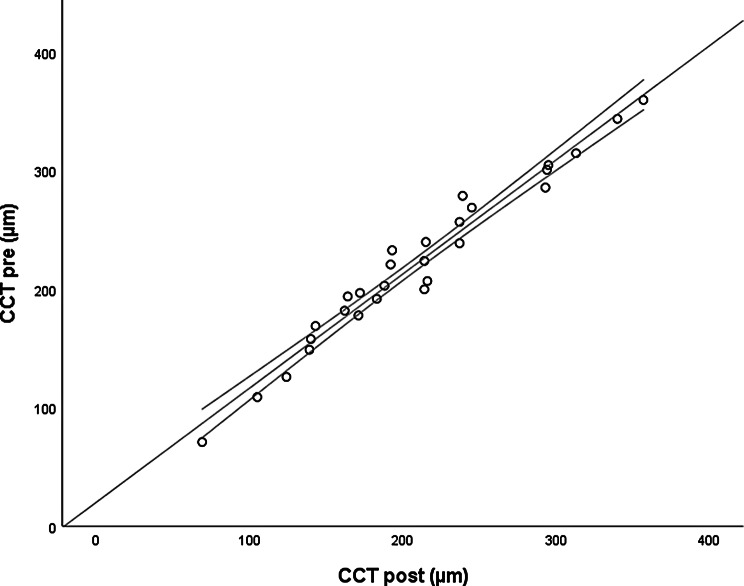
A CCT pre (µm): central choroidal thickness pretreatment, CCT post (µm): central choroidal thickness posttreatment

**Graph. 8:**
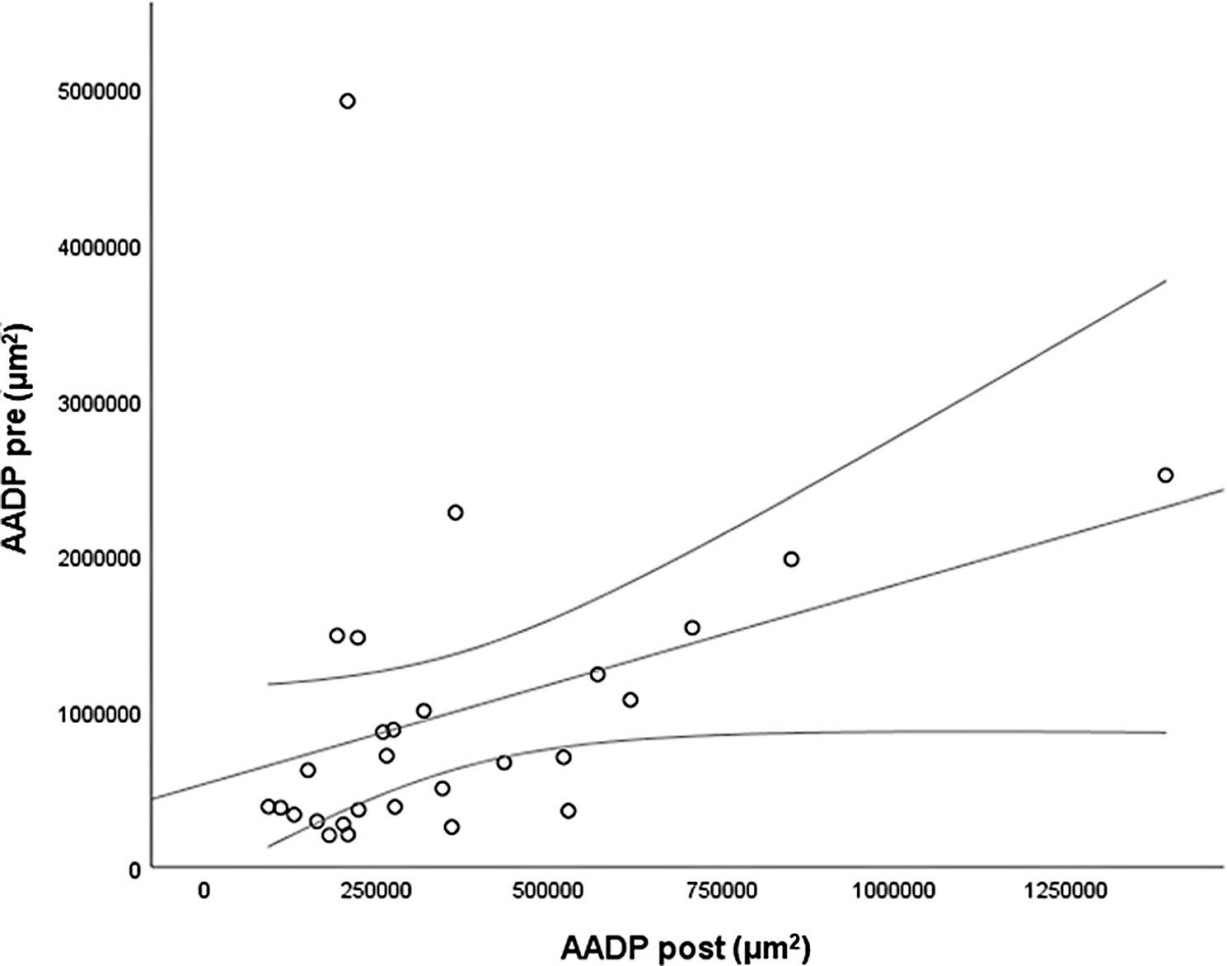
AADP pre (µm^2^): area avascular of the deep plexus pretreatment, AADP post (µm^2^): avascular area of the deep plexus posttreatment

**Graph. 9:**
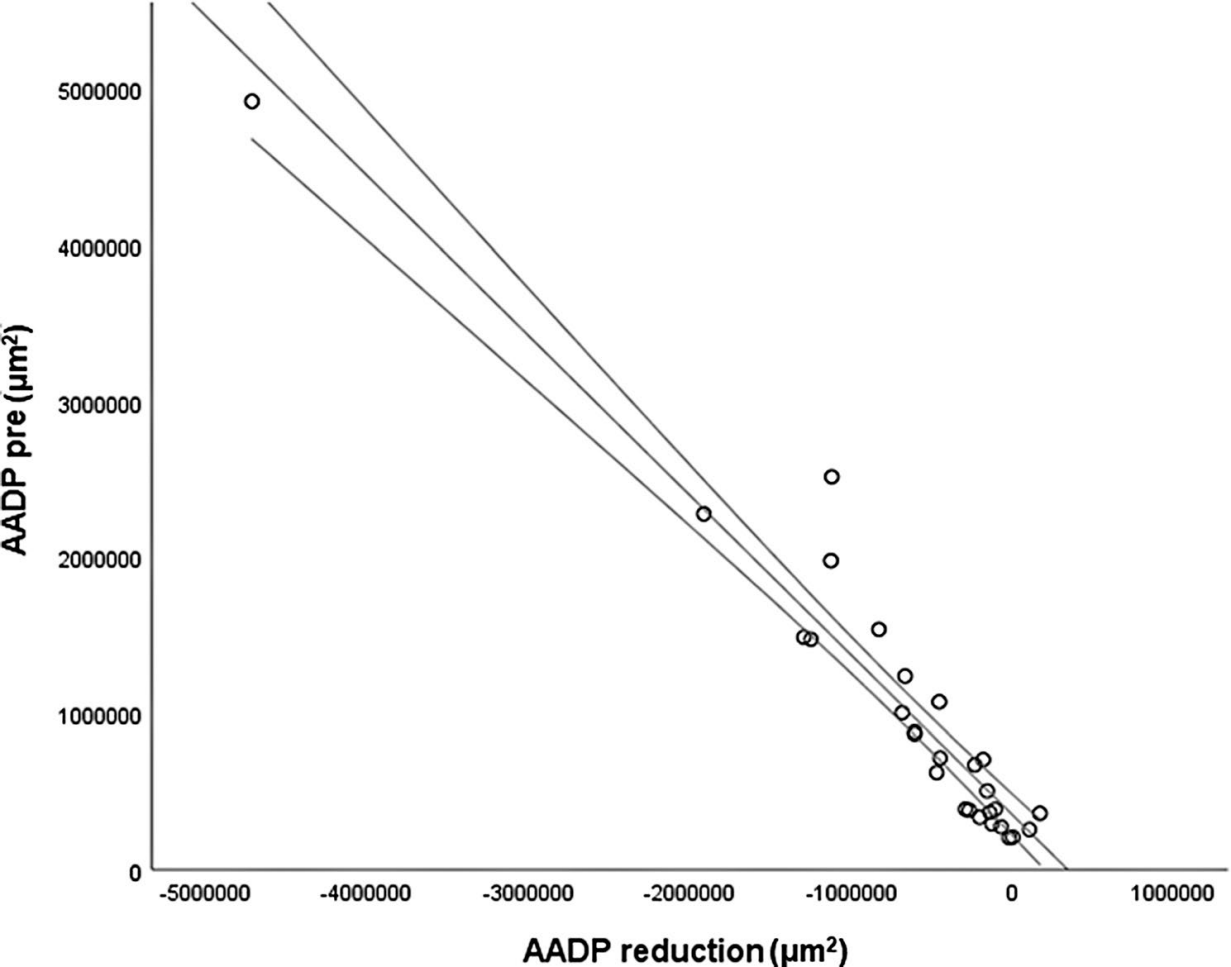
AADP pre (µm^2^): area avascular of the deep plexus pretreatment, AADP reduction (µm^2^): reduction of the avascular area of the deep plexus

**Graph. 10:**
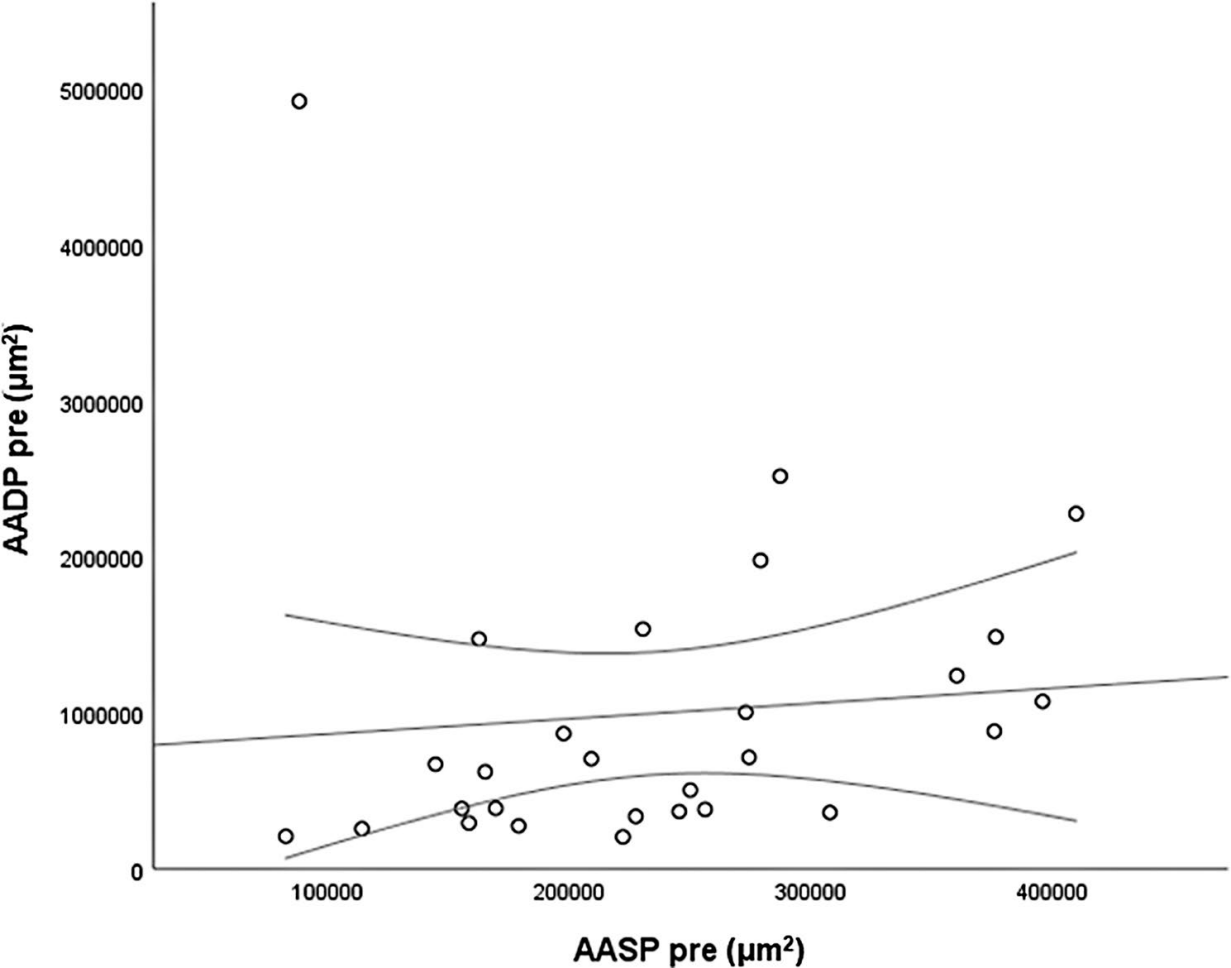
AADP pre (µm^2^): area avascular of the deep plexus pretreatment, AASP pre (µm^2^): avascular area of the superficial plexus pretreatment

**Graph. 11:**
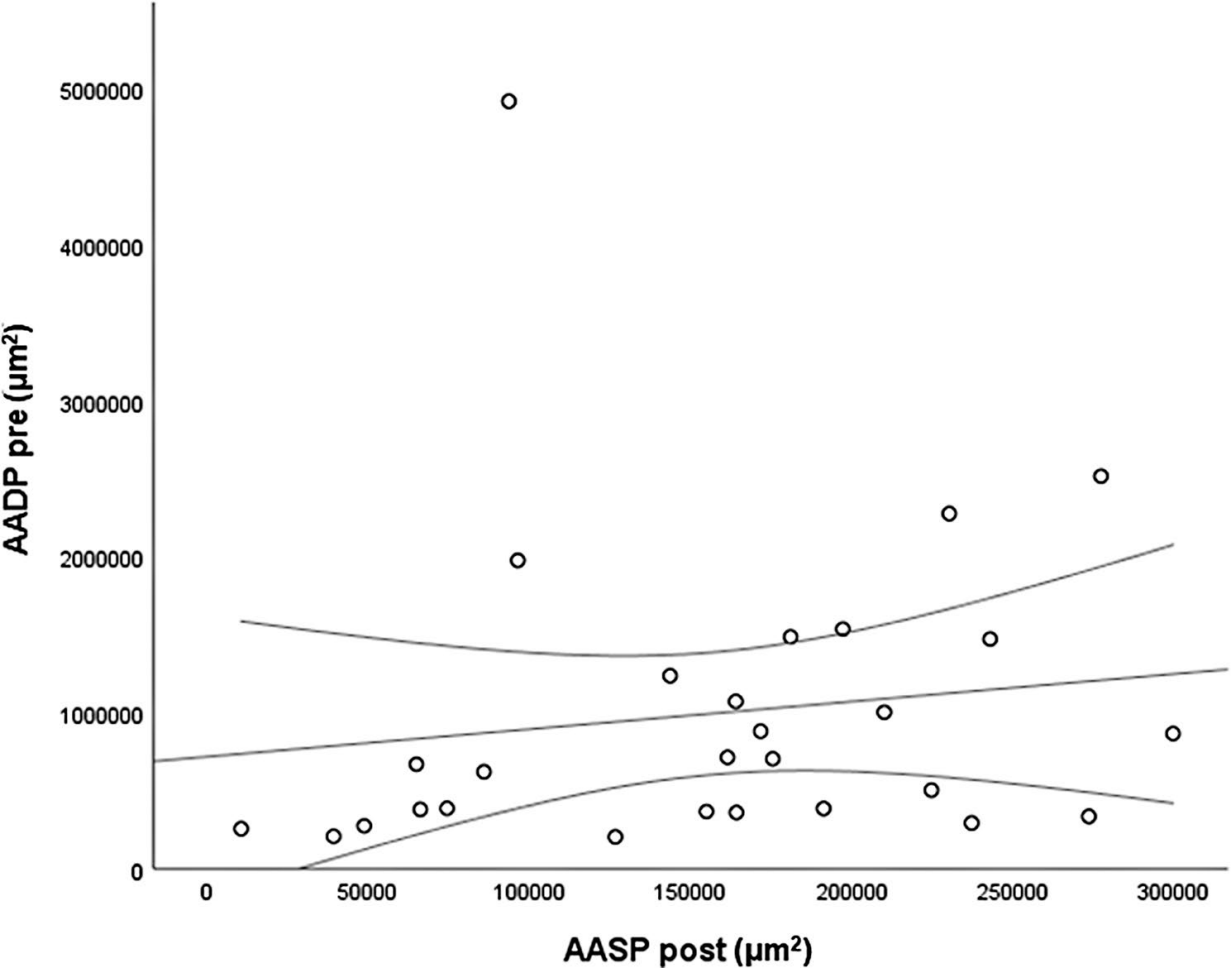
AADP pre (µm^2^): avascular area of the deep plexus pretreatment, AASP post (µm^2^): avascular area of the superficial plexus posttreatment

**Graph. 12:**
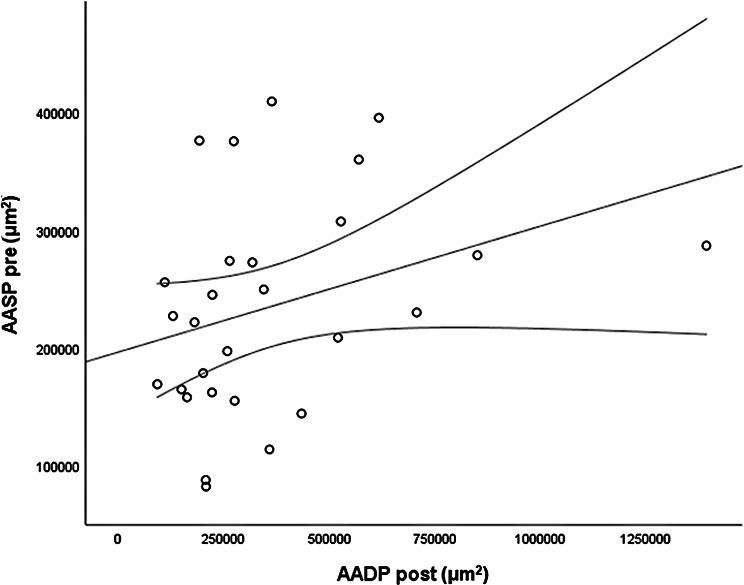
AASP post (µm^2^): avascular area of the superficial plexus pretreatment, AADP post (µm^2^): avascular area of the deep plexus posttreatment

**Graph. 13:**
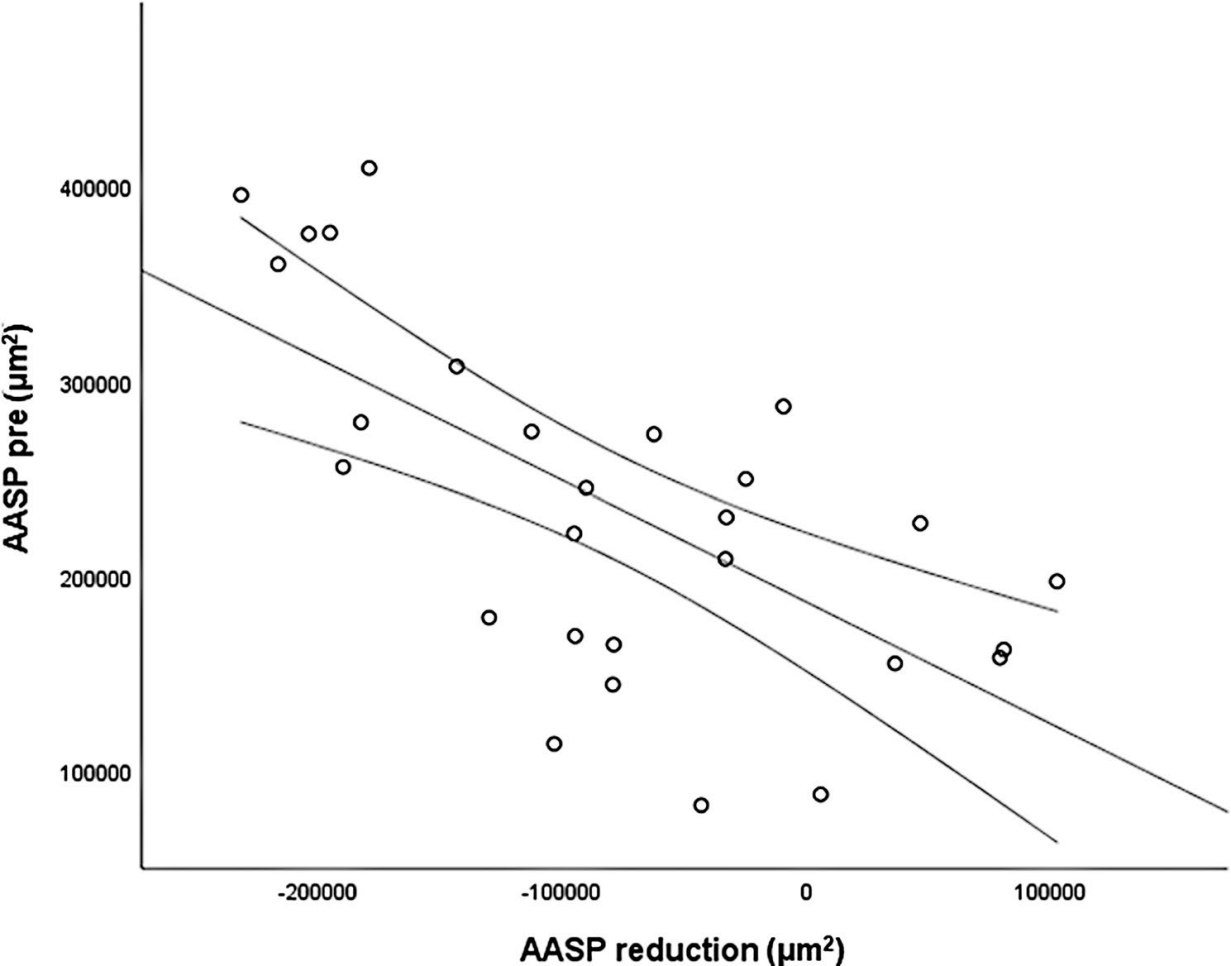
AASP post (µm^2^): avascular area of the superficial plexus pretreatment, AASP reduction (µm^2^): reduction of the avascular area of the superficial plexus

**Graph. 14:**
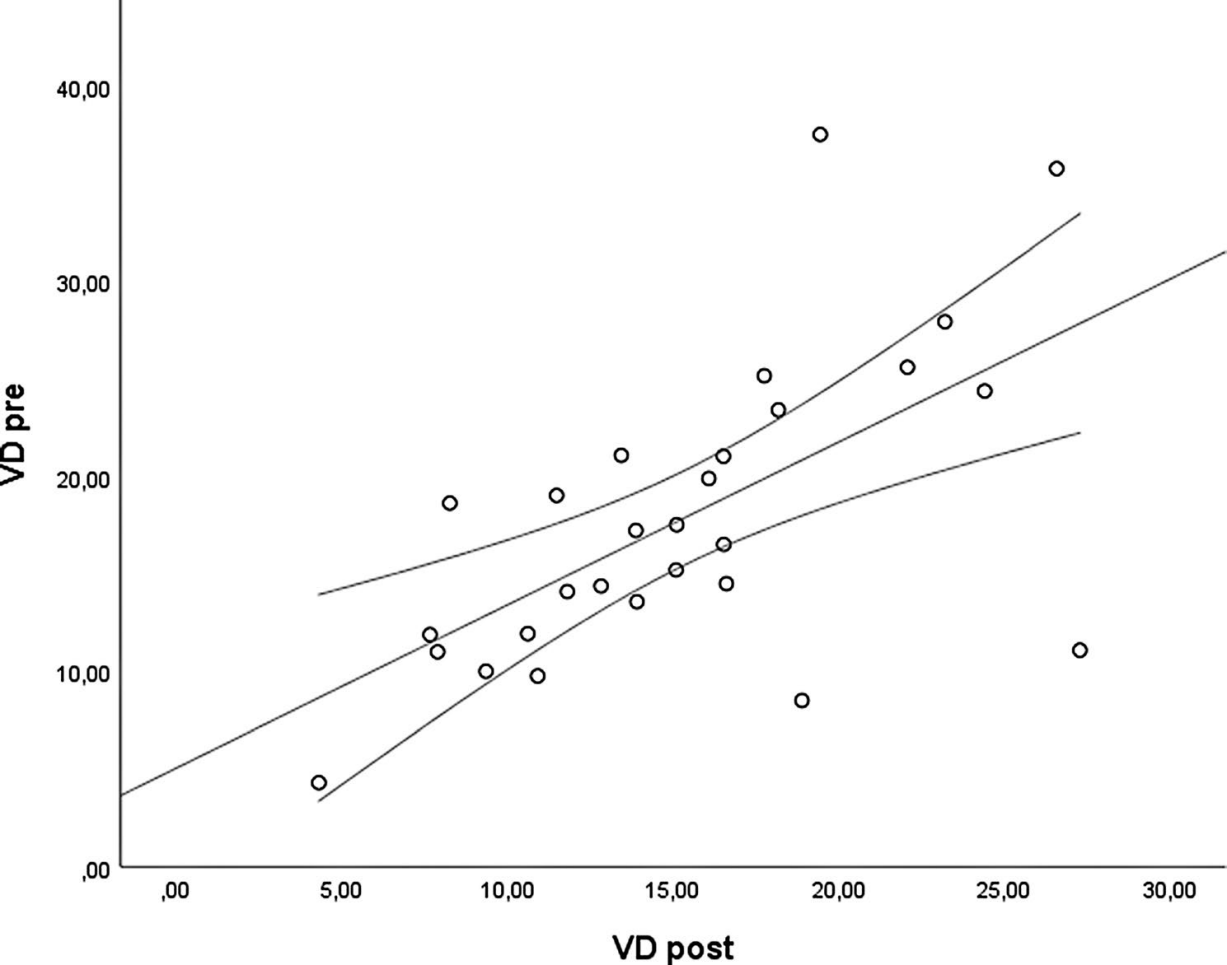
VD pre: vessel density pretreatment, VD post: vessel density posttreatment

**Graph. 15:**
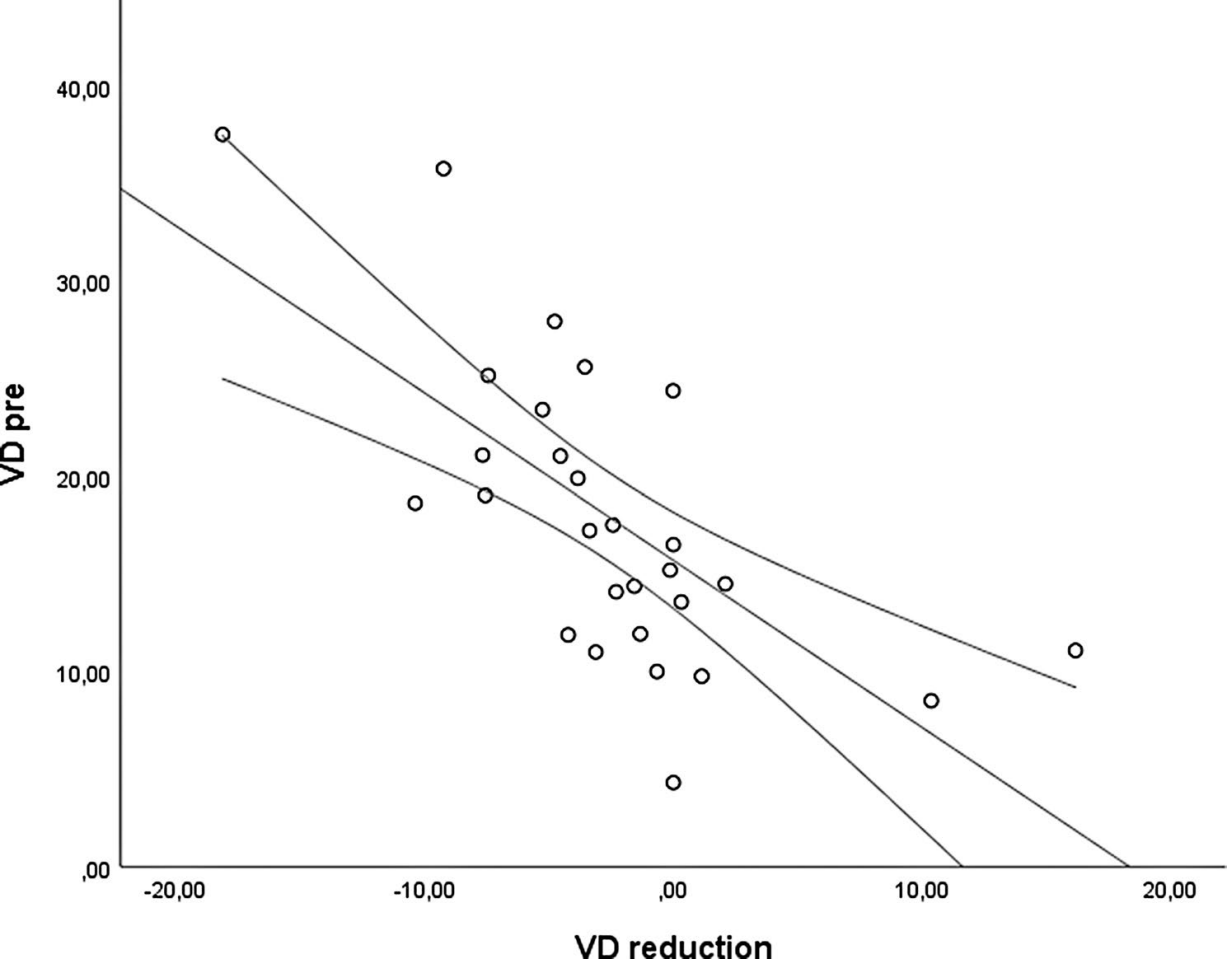
VD pre: vessel density pretreatment, VD reduction: reduction of the vessel density

**Graph. 16:**
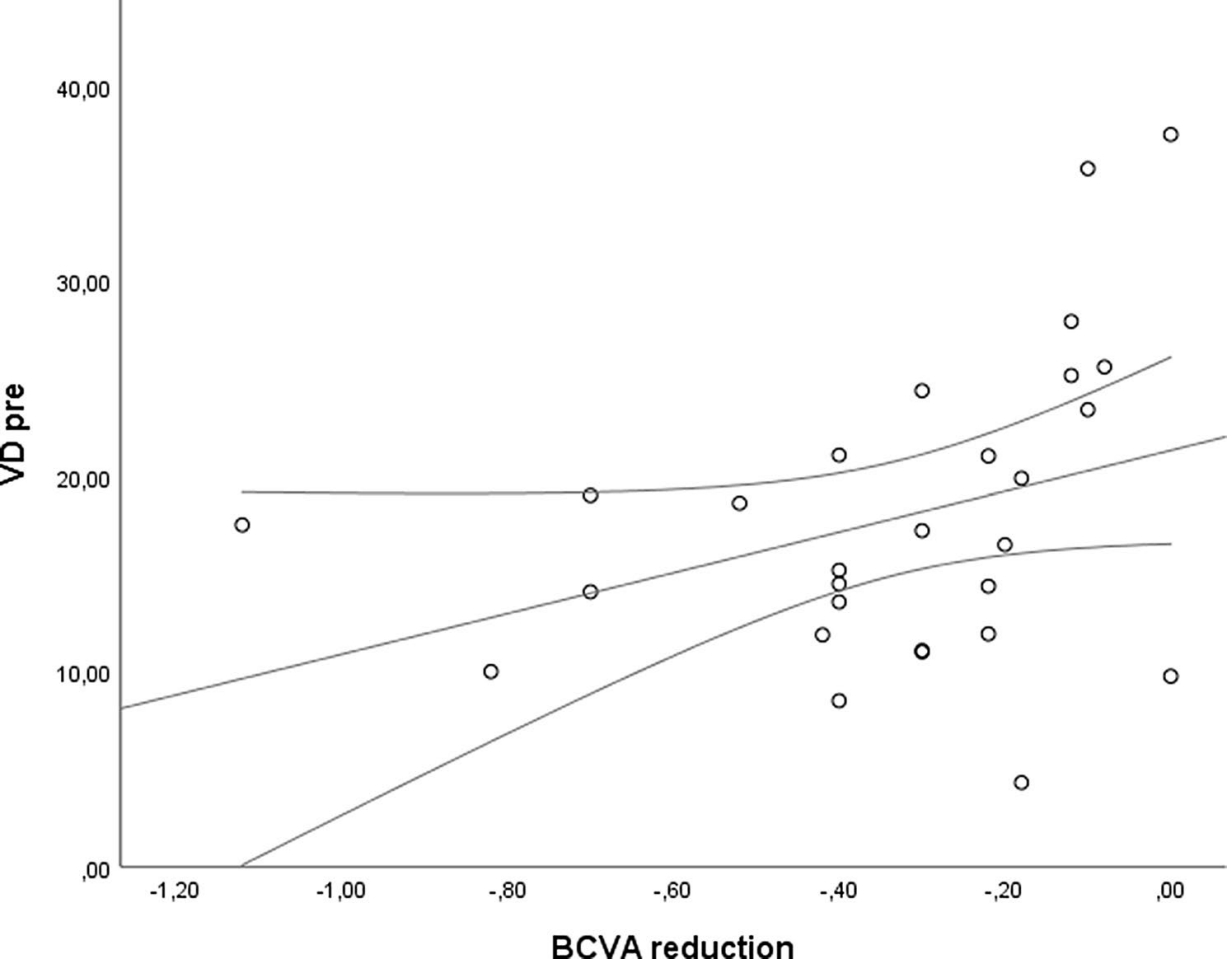
VD pre: vessel density pretreatment, BCVA reduction: reduction of the best-corrected visual acuity

1. The *CMT pre* vs the *CMT post* are significantly correlated (*p* = 0.012), and the correlation is 0.46 (Graph 1), indicating a low positive correlation.
2. The *CMT pre* vs the *CMT reduction* are significantly correlated (*p* < 0.000), and the correlation is −0.61 (Graph 2), indicating a moderate negative correlation.
3. The *CMT pre* vs the *BCVA post* are significantly correlated (*p* = 0.046), and the correlation is 0.38 (Graph 3), indicating a low positive correlation.
4. The *CMT pre* vs the *AADP pre* are significantly correlated (*p* = 0.005), and the correlation is 0.51 (Graph 4), indicating a moderate positive correlation.
5. The *CMT pre* vs the *AADP post* are significantly correlated (*p* = 0.028), and the correlation is 0.41 (Graph 5), indicating a low positive correlation.
6. The *CMT pre* vs the *AADP reduction* are significantly correlated (*p* = 0.011), and it is a correlation of value −0.47 (Graph 6), indicating a low negative correlation.
7. The *CCT pre* vs the *CCT post* are significantly correlated (*p* < 0.000), and it is a correlation of value 0.98 (Graph 7), indicating a very high positive correlation.
8. The *AADP pre* vs the *AADP post* are significantly correlated (*p* = 0.009), and it is a correlation of value 0.48 (Graph 8), indicating a low positive correlation.
9. The *AADP pre* vs the *AADP reduction* are significantly correlated (*p* < 0.000), and it is a correlation of value −0.93 (Graph 9), indicating a very high negative correlation.
10. The *AADP pre* vs the *AASP pre* are significantly correlated (*p* = 0.018), and the correlation is 0.44 (Graph 10), indicating a low positive correlation.
11. The *AADP pre* vs the *AASP post* are significantly correlated (*p* = 0.048), and the correlation is 0.37 (Graph 11), indicating a low positive correlation.
12. The *AASP pre* vs the *AADP post* are significantly correlated (*p* = 0.037), and it is a correlation of value 0.39 (Graph 12), indicating a low positive correlation.
13. The *AASP pre* vs the *AASP reduction* are significantly correlated (*p* < 0.000), and the correlation is −0.65 (Graph 13), indicating a moderate negative correlation.
14. The *VD pre* vs the *VD post* are significantly correlated (*p* < 0.000), and the correlation value is 0.62 (Graph 14), indicating a moderate positive correlation.
15. The *VD pre* vs the *VD reduction* are significantly correlated (*p* < 0.000), and the correlation value is −0.72 (Graph 15), indicating a high negative correlation.
16. The *VD pre* vs the *BCVA reduction* are significantly correlated (*p* = 0.042), and the correlation value is 0.38 (Graph 16), indicating a low positive correlation.

In this way, we had the following: Very high: 2 correlations; High: 1 correlation; Moderate: 4 correlations; and Low: 9 correlations.

Table 5 shows all the correlations evaluated in this study.

## Discussion

This is the first prospective study to comprehensively analyze correlations between retinal and choroidal biomarkers in naïve DME eyes treated with a loading dose of aflibercept using high-resolution SS-OCT and SS-OCTA before treatment and at the 4-month follow-up. While previous studies have described isolated improvements in BCVA, CMT, VD, or choroidal thickness after anti-VEGF therapy [15–17], no studies have explored the correlations among these parameters.

The identification of moderate, high, and very high correlations in our study reinforces the clinical relevance of the observed associations, demonstrating consistent and reproducible relationships between structural and vascular biomarkers in DME. From a clinical standpoint, baseline parameters such as CMT pre, CCT pre, AADP pre, AASP pre, and VD pre may serve not only as descriptors of disease severity but also as potential predictive indicators of therapeutic response. These findings suggest that SS-OCT and SS-OCTA may help clinicians stratify patients more effectively, anticipate the magnitude of anatomical and microvascular improvement, and guide individualized follow-up or retreatment strategies. By showing that several correlations reached moderate to very high levels, our results highlight that these biomarkers are not isolated measures but interconnected parameters that can contribute to more precise and personalized management of DME. This perspective supports the potential value of SS-OCT and SS-OCTA biomarkers within a broader biomarker-based framework for therapeutic monitoring in clinical practice. Future studies with larger cohorts, longer follow-up, and comparative arms involving other anti-VEGF agents are warranted to validate these associations and further clarify their role in clinical decision-making.

It is important to highlight that this study was designed as an exploratory and descriptive correlation analysis, aiming to provide preliminary information on the relationships between structural and vascular biomarkers. Although the identified pairwise correlations show significant descriptive associations, confirmation of independent relationships would require multivariate or partial correlation models in larger datasets.

Although 9 out of the 16 correlations observed in our analysis were classified as low in magnitude, they should not be disregarded. All these associations reached statistical significance and consistently followed biologically plausible directions. It is likely that the relatively small sample size of our study attenuated the strength of some associations; larger cohorts may reveal stronger relationships. As highlighted by Mukaka [11], correlation coefficients should always be interpreted in context, and even weak associations may provide valuable insights when they are systematic and biologically plausible. Moreover, the classification of correlation strength is not universal and may vary according to the scale or thresholds applied, as discussed by Chan [18]. In our study, we adopted a widely accepted metric for interpreting correlation coefficients, which provides a robust and reproducible framework to ensure comparability with other research. It is important to emphasize that this is the first prospective study to systematically evaluate the correlations between SS-OCT and SS-OCTA biomarkers in treatment-naïve eyes with DME treated with a loading dose of aflibercept, thus highlighting the originality and possible practical utility of these findings.

It is important to emphasize that our cohort included eyes with moderate and severe non-proliferative DR. However, due to the limited sample size and the predominance of severe non-proliferative DR cases, a stratified analysis according to the severity of DR or other covariates was not feasible. This aspect should be addressed in future studies with larger and more balanced cohorts.

Previous studies have demonstrated that intravitreal aflibercept reduces CMT and improves VA, and our findings are consistent with these observations [19–21].

We focused mainly on the biomarkers for which we found statistical correlations:

A-) Very high: 1-) *CCT pre* and the *CCT post*; and

2-) *AADP pre* and the *AADP reduction*;

B-) High: 3-) *VD pre* and the *VD reduction*.

### CCT

Among healthy individuals, choroidal thickness is typically reported to range from 200 to 300 µm. Exact values can differ slightly across populations and according to the imaging protocol or segmentation approach employed [22, 23 ]. A number of studies in distinct cohorts corroborate this interval as a practical reference range, which is useful when evaluating choroid-related clinical measurements [22, 23 ].

In our study, the correlation between *CCT pre* and *CCT post* reached 0.98 (*p* < 0.000), which is classified as a very high positive correlation, according to the classification proposed by Mukaka [11]. This result demonstrates the existence of an extremely stable linear relationship between these measurements, reinforcing the statistical robustness of the findings and reducing the possibility that the results were attributable to chance. From a clinical perspective, the high correlation observed indicates that the CCT is a stable structural biomarker: eyes with thicker choroids at baseline remained relatively thicker after therapy, regardless of the mean reduction observed. Previous studies have described significant reductions in choroidal thickness in patients with DME treated with intravitreal therapies, such as aflibercept [24] and bevacizumab [25], as well as the association between different stages of DR and choroidal thinning [26]. However, no previous study has reported a high positive correlation between *CCT pre* and *CCT post* values in treatment-naïve patients. Therefore, the CCT should be evaluated not only in absolute terms (reduced or not reduced) but also in relative terms, since each patient maintains their “choroidal profile” even after treatment. Thus, the CCT can serve as a reliable and consistent marker for the longitudinal follow-up of patients with DME receiving intravitreal therapy with aflibercept.

### AADP

We observed a very strong negative correlation between *AADP pre* and the *AADP reduction* after treatment (*r* = −0.93; *p* < 0.000). This high correlation highlights the potential of baseline AADP as a prognostic biomarker, allowing clinicians to predict which patients are likely to exhibit greater microvascular reperfusion after aflibercept. This finding indicates that eyes with higher baseline AADP tend to show more pronounced shrinkage of this area with treatment. Statistically, this correlation reinforces the predictability and reliability of the therapeutic effect on the microvasculature in DME patients. Previous studies have shown that compared with eyes without edema, naïve patients with DME exhibit larger areas of capillary nonperfusion in the deep plexus (Braham et al., 2022) [27], as well as an association between deep plexus perfusion and retinal thickness changes after edema resolution (Moon et al., 2018) [28]. However, to our knowledge, no study has reported such a strong negative correlation between baseline AADP and its posttreatment reduction. Clinically, these findings suggests that baseline AADP may also function as a robust prognostic biomarker, allowing prediction of the magnitude of response, and helps to understand that quantitative assessment of AADP may be useful in the follow-up of patients with DME receiving aflibercept therapy.

### VD

VD is defined as the percentage of the scan area occupied by perfused vessels and was one of the earliest quantitative outputs from OCTA angiograms [29, 30]. In this study, the total retinal VD was significantly lower at the visit after the loading phase than at baseline. To our knowledge, this has not been shown previously when analyses were restricted to patients with naïve DME treated with an aflibercept loading dose and imaged with SS-OCT and SS-OCTA. These findings contrasts with those of Conti et al., who detected no change between pre and posttreatments assessments [31]. Santamaria et al. 2024 described a downward trend after six months of anti-VEGF treatment that did not meet their statistical cutoff [32]. In line with this decrease, Kansal et al. reported reduced VD after five injections of aflibercept, but only in eyes categorized as responders [33]. These varied outcomes illustrate challenges in VD quantification because edema-related artifacts can destabilize segmentation across visits and macular exudation may suppress signals, both of which can affect the estimates [34]. Therefore, the observed reduction in VD in our cohort should be interpreted with caution, since segmentation instability, projection artifacts, and edema-related shadowing can influence quantitative estimates. In addition, heterogeneity in methodological approaches across OCTA studies (including differences in scan size, slab definitions, and post-processing algorithms) further complicates direct comparison of VD outcomes in the literature.

In our study, we observed a reduction in VD, in contrast to the findings of Alagorie et al. [34], who reported that VD did not change after 12 months of intravitreal aflibercept therapy. However, there are some differences in methodology between the studies, such as monthly intravitreal injection of aflibercept 2 mg, and the patients did not have DME but rather had proliferative DR. This difference highlights the complexity of the study of these patients.

In our investigation, *VD pre* showed a strong negative correlation with its own reduction after treatment (*r* = −0.72; *p* < 0.000). These findings indicate that eyes with higher baseline VD tend to experience more pronounced decreases after treatment, whereas eyes with lower baseline VD show less significant changes. Statistically, this pattern reinforces the consistency of the findings and demonstrates that the microvascular response does not occur randomly, but rather follows a predictable gradient depending on the baseline value. From a clinical perspective, the correlation found suggests that VD may act as a marker of therapeutic response, as higher baseline levels are associated with a greater magnitude of posttreatment reduction. Previous OCTA studies have reported changes in VD after DME treatment, with reduced VD in the superficial and deep retina after anti-VEGF treatment, albeit with heterogeneous results [24, 35]. However, no previously published studies have shown a statistically significant correlation. Thus, our findings are unique and reinforce the potential of baseline VD as a prognostic biomarker capable of predicting the magnitude of the vascular response in patients with DME receiving aflibercept treatment.

Limitations: Our study has some limitations, such as the single-center design and the relatively small sample size. Although SS-OCTA provides a high-resolution assessment of the retinal microvasculature, it remains sensitive to segmentation errors and motion artifacts that can affect quantitative measurements. Furthermore, the analyses were exploratory and based solely on bivariate correlations, without adjustment for potential collinearity or confounding factors. Future studies employing multivariate or partial correlation approaches are warranted to validate the independence and robustness of the observed associations. Additionally, analyses stratified by DR severity or other covariates were not feasible due to the limited sample size and should be conducted in larger multicenter cohorts. Moreover, the 4-month follow-up period limits the ability to determine whether the observed correlations persist over longer intervals, and prolonged follow-up would be valuable to assess the temporal stability of these biomarker relationships (although the initial objective of this study was a 4-month follow-up). Despite these limitations, this study provides novel insights into aflibercept treatment for DME in treatment-naïve patients based on the comprehensive evaluation of SS-OCT and SS-OCTA biomarkers.

## Conclusion

This is the first study to investigate correlations between SS-OCT and SS-OCTA biomarkers before and after a loading phase of three monthly aflibercept injections in treatment-naive patients with DME, over a 4-month follow-up period. Our findings contribute to a better understanding of the relationships between retinal and choroidal biomarkers in the treatment of DME. Thus, our study provides valuable insights into the role of these baseline biomarkers and supports the use of the SS-OCT and SS-OCTA measurements as objective parameters for anticipating treatment responses and for guiding individualized treatment plans to optimize DME management. Future multicenter studies with larger sample sizes and longer follow-up periods are needed to confirm these findings.

## Data Availability

All datasets presented in the study are included in the article/Supplementary Material.

## Abbreviations

AADP: Avascular area of the deep plexus
AASP: Avascular area of the superficial plexus
BCVA: Best-corrected visual acuity
CAAE: Certificado de Apresentação de Apreciação Ética (Ethics Committee Identifier, Brazil)
CCT: Central choroidal thickness
CMT: Central macular thickness
CVI: Choroidal vascularity index
DCP: Deep capillary plexus
DM: Diabetes mellitus
DME: Diabetic macular edema
DR: Diabetic retinopathy
DRCR.net: Diabetic Retinopathy Clinical Research Network
EDI: Enhanced depth imaging
ETDRS: Early Treatment Diabetic Retinopathy Study
FA: Fluorescein angiography
FAF: Fundus autofluorescence
FAZ: Foveal avascular zone
logMAR: Logarithm of the minimum angle of resolution
OCT: Optical coherence tomography
OCTA: Optical coherence tomography angiography
PED: Pigment epithelium detachment
PRN: Pro re nata (as needed)
RPE: Retinal pigment epithelium
SCP: Superficial capillary plexus
SD-OCT: Spectral-domain optical coherence tomography
SS: Swept-source
SS-OCT: Swept-source optical coherence tomography
SS-OCTA: Swept-source optical coherence tomography angiography
VA: Visual acuity
VD: Vessel density
